# Poly(ADP-ribose) polymerase 1 inhibition protects cardiomyocytes from inflammation and apoptosis in diabetic cardiomyopathy

**DOI:** 10.18632/oncotarget.8343

**Published:** 2016-03-24

**Authors:** Wei-dong Qin, Guo-liang Liu, Juan Wang, Hao Wang, Jian-ning Zhang, Fan Zhang, Yang Ma, Xin-ying Ji, Chen Li, Ming-xiang Zhang

**Affiliations:** ^1^ Department of Critical Care Medicine, Qilu Hospital of Shandong University, Jinan, Shandong, China; ^2^ The Henan Provincial Key Engineering Laboratory of Antibody Drugs, School of Medicine, Henan University, Kaifeng, Henan, China; ^3^ Department of Cardiology, The Second Hospital of Shandong University, Jinan, Shandong, China; ^4^ The Key Laboratory of Cardiovascular Remodeling and Function Research, Chinese Ministry of Education and Chinese Ministry of Public Health, Qilu Hospital of Shandong University, Jinan, Shandong, China

**Keywords:** diabetic cardiomyopathy, poly(ADP-ribose) polymerase 1, hyperglycemia, inflammatory response, apoptosis, Pathology Section

## Abstract

Diabetic cardiomyopathy (DCM) is characterized by structural alterations such as cardiomyocyte hypertrophy, necrosis and focal fibrosis. Poly(ADP-ribose) polymerase 1 (PARP-1) is a nuclear enzyme which can be activated by DNA damage and plays a critical role in various diseases. We hypothesized that PARP-1 may play an important role in DCM and that its inhibition may protect cardiomyocytes from inflammation and apoptosis in DCM. H9c2 cardiomyocytes were treated with normal glucose, mannitol or high glucose (HG). Male C57BL/6 mice or PARP-1−/− mice were treated with streptozotocin (STZ) by intraperitoneal injection for 5 consecutive days to induce diabetes. In vitro, HG stimulation induced oxidative stress and DNA damage and increased PARP-1 expression and activity. Compared with the control, pretreatment with PARP-1 siRNA significantly reduced HG-induced inflammatory response, including tumor necrosis factor-α (TNF-α), interleukin-1β (IL-1β) and IL-6 secretion, and intercellular adhesion molecule-1 (ICAM-1) and inducible nitric oxide synthase (iNOS) expression. PARP-1 inhibition reduced HG-induced cardiomyocyte apoptosis through downregulation of cleaved caspases and activation of IGF-1R/Akt pathway. In vivo, hyperglycemia increased the protein expression of nitrotyrosine and PARP-1 as well as PARP-1 activity. PARP-1 gene deletion significantly improved cardiac dysfunction and reduced inflammatory response and apoptosis. This work demonstrated the critical role of PARP-1 in diabetic heart injury, and suggested that PARP-1 inhibition may be a feasible strategy for the treatment of DCM.

## INTRODUCTION

Diabetes mellitus (DM) is a chronic metabolic disorder manifested by a loss of pancreatic islet B cell, decreased serum insulin, and hyperglycemia [1]. Diabetic cardiomyopathy (DCM), manifested by left ventricle diastolic and systolic dysfunction, is one of the most common causes of morbidity and mortality in DM [2].

Inflammation with increased cytokine levels played an important role in the pathogenic development of DCM [3, 4]. Over-production of pro-inflammatory cytokines, such as tumor necrosis factor-α (TNF-α) and interleukin-1β (IL-1β), could stimulate the expression of inflammatory mediators as a positive feedback mechanism and induce cardiomyocyte apoptosis, which eventually result in cardiac dysfunction [5]. Apoptosis of cardiomyocytes is one of the most important outcomes of hyperglycemia-induced inflammation and oxidative stress in the heart [6]. Increased cardiomyocyte apoptosis has been reported in diabetic animal models and patients as a predominant cause for the loss of contractile tissues, remodeling, and eventually dysfunction [7–9]. Sustained inflammation may lead to the activation of multiple pathways that lead to cell death [10, 11]. TNF-α has been demonstrated to provoke cardiomyocytes apoptosis and cardiac remodeling through activation of multiple cell-death pathways [12, 13]. Cardiomyocyte death results in a loss of contractile tissue and initiates a cardiac remodeling [14].

Hyperglycemia induced oxidative stress and nitrosative stress, related to the formation of reactive oxygen and nitrogen species, most notably peroxynitrite, plays a critical role in cardiomyocyte apoptosis in diabetes [15, 16]. Peroxynitrite, formed from the reaction of nitric oxide (NO) and superoxide anion (O_2_^−^), can induce DNA strand breaks, protein modifications and alterations in cell signaling [17]. Administration of antioxidant agents is able to rescue cardiomyocytes. The mechanisms activated by hyperglycemia, leading to myocardial oxidative stress and apoptosis, are not fully investigated.

The nuclear enzyme poly(ADP-ribose)polymerase 1 (PARP-1), the most abundant isoform of the PARP enzyme family, has been implicated in the regulation of multiple physiological cellular functions such as DNA repair, gene transcription, cell death, and genomic stability [18]. As a DNA damage sensor, PARP-1 can be activated by damaged DNA and catalyzes the cleavage of NAD^+^ into nicotinamide and ADP-ribose to form long branches of ADP-ribose polymers on target proteins [17]. Excessive activation of this enzyme induces the intracellular depletion of NAD^+^ and ATP, resulting in a cellular energy crisis and irreversible cytotoxicity, even cell death [19]. PARP-1 can also regulate the expression of a variety of key inflammatory genes, such as inducible nitric oxide synthase (iNOS), intercellular adhesion molecule-1 (ICAM-1), and vascular cell adhesion molecule-1 (VCAM-1), all of which are regulated by nuclear factor-κB (NF-κB) [20]. Overactivation of PARP-1 is an important mechanism leading to tissue damage in various pathological conditions associated with oxidative stress, including myocardial reperfusion injury, stroke, and shock, while PARP-1 inhibitors showed pronounced protection against these diseases [21–23]. PARP-1 inhibition can limit cellular energy depletion and protect cells from death. Moreover, recent evidence suggests that PARP-1 inhibition can activate a prosurvival signaling cascade through Akt phosphorylation [24].

The insulin-like growth factor 1 receptor (IGF-1R) has surfaced as a significant target in multiple solid cancers due to its fundamental role in pro-survival and anti-apoptotic signaling [4]. Overexpression of the insulin-like growth factor 1 (IGF-1) can decrease cardiomyocytes death [14], *via* activation of the IGF-1 receptor (IGF-1R), and subsequent activation of the PI3K/Akt pathway [25]. In the present study, we investigated the role and underlying mechanism of PARP-1 in hyperglycemia induced DCM. Our data demonstrated that PARP-1 inhibition significantly reduced cardiac inflammation, apoptosis, and dysfunction associated with DCM. The cardiac protection from diabetes, including anti-inflammation and anti-apoptosis, was found to be partly mediated by activation of IGF-1R/Akt pathway.

## RESULTS

### HG increased PARP-1 expression and activity and induced DNA damage

**Figure 1:**
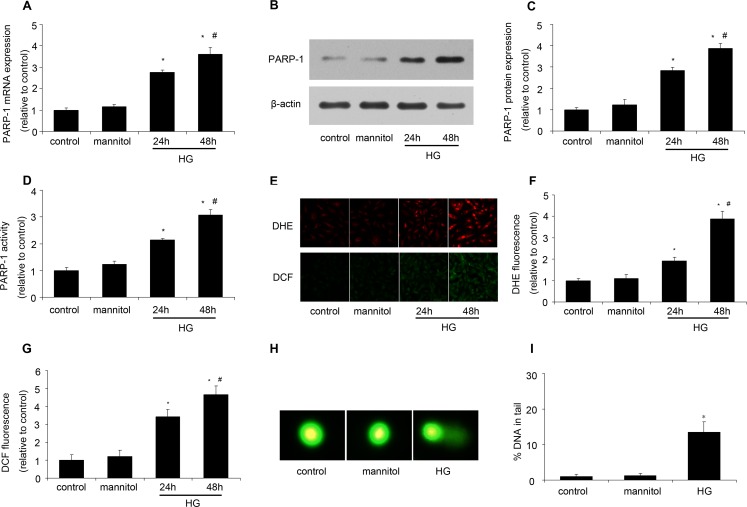
HG increased PARP-1 expression and activity and induced DNA damage After H9c2 cells were stimulated by normal glucose (control, 5.5 mmol/l glucose), mannitol (5.5 mmol/l glucose plus 24.5 mmol/l mannitol) or high glucose (HG, 33mmol/l glucose), PARP-1 expression and activity was assessed, oxidative stress was determined by DHE and DCF staining, and DNA damage was assessed by comet assay. **A.** HG simulation increased PARP-1 mRNA expression as determined by RT-PCR. **B.**, **C.** HG simulation increased PARP-1 protein expression as assessed by western blotting analysis. **D.** HG simulation increased PARP-1 activity as determined by spectrophotometer. **E.**, **F.**, **G.** HG simulation induced oxidative stress. **H.**, **I.** HG treatment induced DNA damage. Values are expressed as mean ± S.D. from three independent experiments. *P < 0.05 *vs*. control. ^#^*P* < 0.05 *vs*. 24h. HG: high glucose.

After H9c2 cells were stimulated by mannitol (5.5 mmol/l glucose plus 24.5 mmol/l mannitol) or HG (33mmol/l glucose), PARP-1 expression and activity were assessed in our experiment. As shown in Figure 1, compared with the control (5.5 mmol/l glucose), mannitol had no effect on PARP-1 expression and activity, while HG could increase the expression and activity of PARP-1 in a time dependent manner (Figure 1A-1D). Then we investigated the underlying mechanism. As a DNA damage sensor, PARP-1 can be activated by damaged DNA. Thus, we assessed oxidative stress by DHE and DCF staining and DNA damage by comet assay. We found that HG simulation could significantly increase oxidative stress and DNA damage, while mannitol had no effect on them (Figure 1E-1I). These results suggested that HG could increase PARP-1 expression and activity and induce DNA damage.

### PARP-1 inhibition alleviated HG-increased inflammatory response *in vitro*

**Figure 2:**
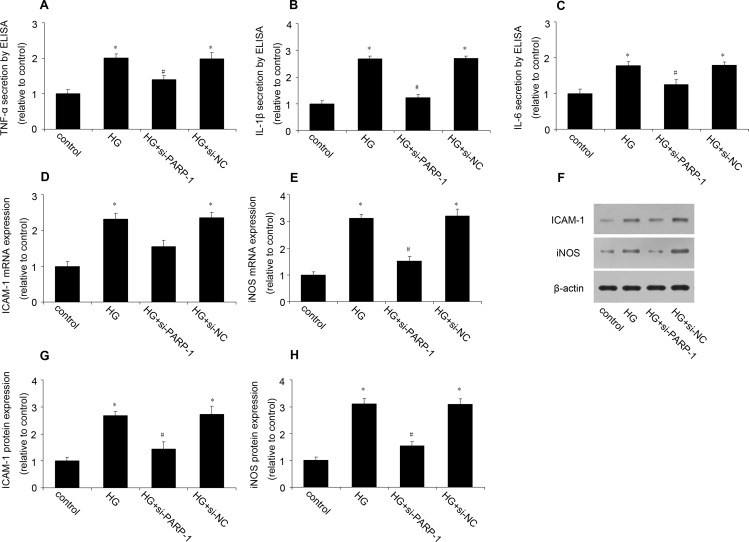
PARP-1 inhibition reduced HG-induced inflammatory response H9c2 cells were stimulated by normal glucose or HG after PARP-1 was inhibited by siRNA for 24hrs. The secretion of TNF-α, IL-1β and IL-6 was determined by ELISA, the expression of ICAM-1 and iNOS was assessed by RT-PCR and western blotting analysis. **A.**, **B.**, **C.** PARP-1 inhibition by siRNA reduced HG-induced the cellular secretion of TNF-α, IL-1β and IL-6. **D.**, **E.** PARP-1 inhibition reduced HG-induced the mRNA expression of ICAM-1 and iNOS. **F.**, **G.**, **H.** PARP-1 inhibition reduced HG-induced the protein expression of ICAM-1 and iNOS. Values are expressed as mean ± S.D. *P < 0.05 *vs*. control. ^#^*P* < 0.05 *vs*. HG. HG: high glucose; si-PARP-1: PARP-1 siRNA; si-NC: negative control of PARP-1 siRNA.

To investigate the role of PARP-1 in HG-induced inflammation in cardiac cells, H9c2 cells were treated with PARP-1 siRNA for 24h, and then simulated by HG for 48h. We firstly validated the efficiency of PARP-1 siRNA. RT-PCR and western blotting analysis shown that PARP-1 siRNA could significantly reduce PARP-1 mRNA and protein expression, while the negative control of siRNA (si-NC) had no effect on it (Supplementary Figure 2). As shown in Figure 2A-2C, ELISA showed that HG treatment significantly increased the secretion of TNF-α, IL-1β and IL-6, which were remarkably inhibited by pretreatment with PARP-1 siRNA. The secretion of cytokines was not affected by mannitol, indicating that the osmotic effect did not induce inflammation. PARP-1 siRNA also decreased HG-induced ICAM-1 and iNOS expression, while si-NC had no effect on them (Figure 2D-2H). These results suggested that PARP-1 inhibition could reduce HG-induced inflammatory response in cells.

### PARP-1 inhibition reduced HG-induced cell apoptosis

**Figure 3:**
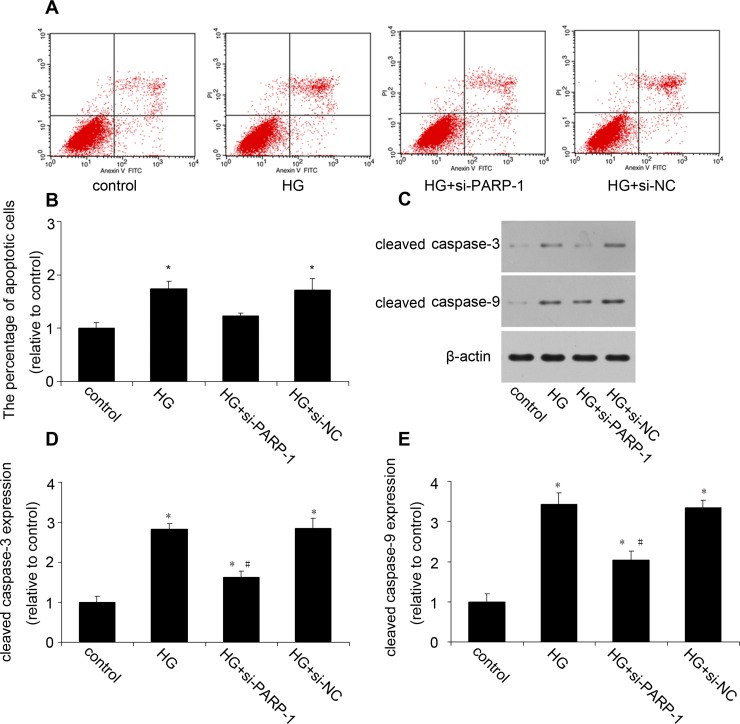
PARP-1 inhibition reduced HG-induced cell apoptosis H9c2 cells were stimulated by normal glucose or HG after PARP-1 was inhibited by siRNA for 24hrs. Cell apoptosis was assessed by flow cytometry and cleavaged caspase was detected by western blotting analysis. **A.**, **B.** PARP-1 inhibition reduced HG-induced cell apoptosis. **C.**, **D.**, **E.** PARP-1 inhibition reduced HG-upregulated the protein expression of cleaved caspase-3 and caspase-9. Values are expressed as mean ± S.D. *P < 0.05 *vs*. control. ^#^*P* < 0.05 *vs*. HG. HG: high glucose; si-PARP-1: PARP-1 siRNA; si-NC: negative control of PARP-1 siRNA.

We then examined the effect of PARP-1 inhibition on HG-induced cardiac apoptosis. The flow cytometry showed that HG induced a significant increase in cardiac cell apoptosis, which was attenuated by PARP-1 siRNA (Figure 3A and 3B). Then we investigated the probable mechanism. Figure 3C to 3E showed that HG increased the protein expression of cleaved caspase-3 and caspase-9, while PARP-1 inhibition could reduce their expression.

### PARP-1 inhibition increased IGF-1R/Akt phosphorylation *in vitro*

**Figure 4:**
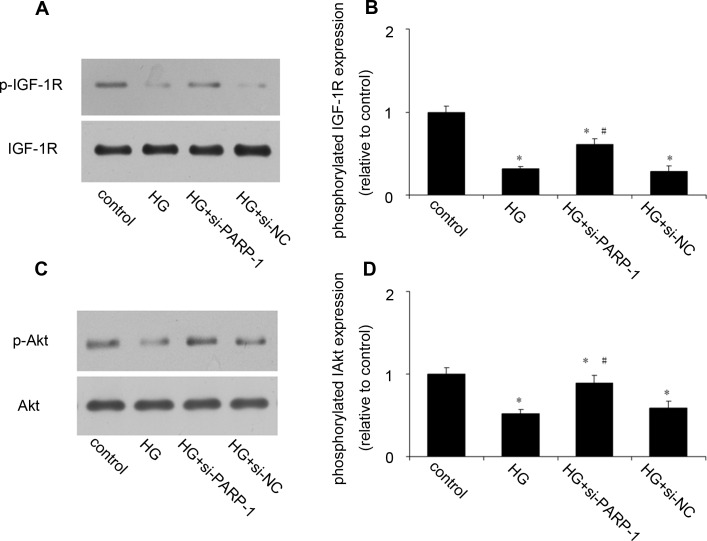
PARP-1 inhibition increased HG-reduced the phosphorylation of IGF-1R/Akt H9c2 cells were stimulated by normal glucose or HG after PARP-1 was inhibited by siRNA for 24hrs. The protein expression of phosphorylated IGF-1R and Akt was detected by western blotting analysis. **A.**, **B.** PARP-1 inhibition increased HG-reduced the phosphorylation of IGF-1R. **C.**, **D.** PARP-1 inhibition increased HG-reduced the phosphorylation of Akt. Values are expressed as mean ± S.D. *P < 0.05 *vs*. control. ^#^*P* < 0.05 *vs*. HG. HG: high glucose; si-PARP-1: PARP-1 siRNA; si-NC: negative control of PARP-1 siRNA.

Then we investigated the probable survival mechanism of PARP-1 inhibition. As we all know, Akt activation plays a critical role in cell survival. PARP-1 inhibition has been demonstrated to increase Akt phosphorylation in neuronal and liver cells exposed to oxidative stress [24]. Cardiac overexpression of IGF-1R prevented diabetes-induced cardiac fibrosis and diastolic dysfunction [27]. In our experiment, compared with the control, HG treatment had no effect on total IGF-1R and Akt expression, but it significantly reduced the phosphorylation of IGF-1R and Akt, while PARP-1 inhibition could increase IGF-1R/Akt phosphorylation (Figure 4). Further experiment was needed to investigate the precise mechanism.

### PARP-1 deletion improved cardiac function in DCM

**Figure 5:**
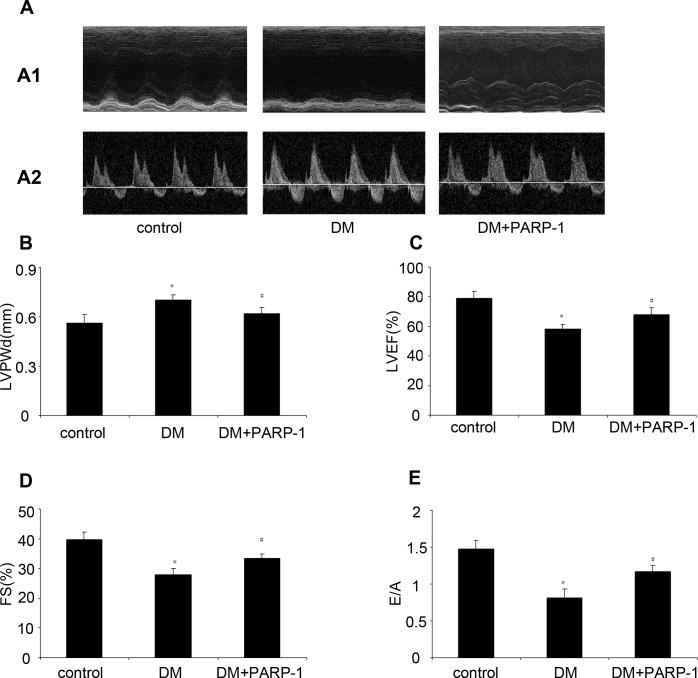
PARP-1 inhibition improved hyperglycemia-reduced cardiac function Before mice were sacrified, cardiac diameter and function were measured by Echocardiography. Compared with the control, hyperglycemia increased LVPWd and reduced LVEF, FS and E/A in DM mice, while PARP-1 deletion alleviated these effects. LV: Left ventricular; LVEF: left ventricular ejection fraction; FS: fractional shortening; E/A: ratio of early to late mitral inflow velocity; LVEDd: left ventricular end-diastolic dimension. Values are expressed as mean ± S.D. *P < 0.05 *vs*. control. DM: diabetic cardiomyopathy.

The ventricular functions of mice were assessed by echocardiography before sacrifice. Compared with the normal control mice, there was a decrease of LVEF, FS and E/A in DM mice, while LVPWd was increased. However, PARP-1 gene deletion could improve the cardiac function, which suggested a protective role of PARP-1 in cardiac function during DCM (Figure 5).

### PARP-1 deletion alleviated hyperglycemia induced heart remodeling

**Figure 6:**
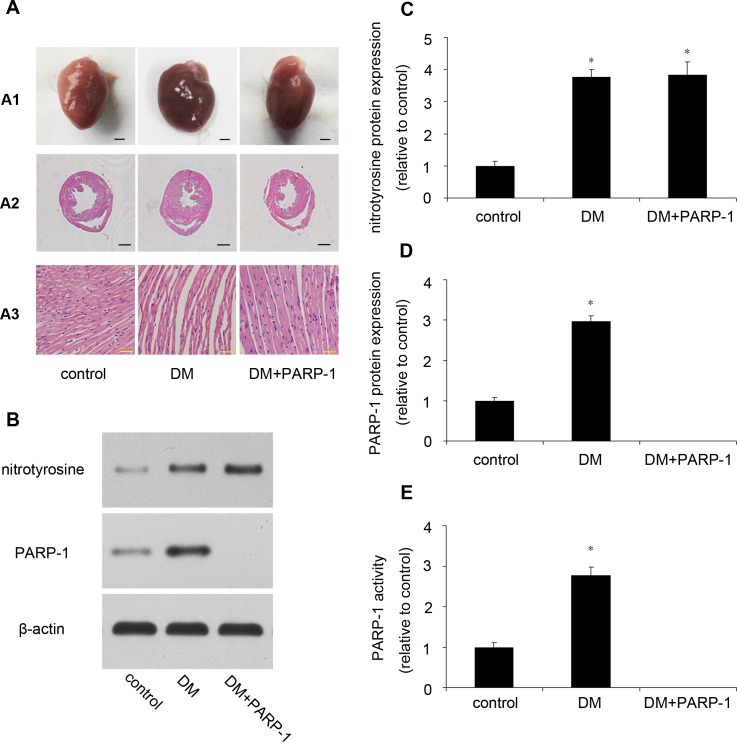
Myocardial pathology feature and PARP-1 expression in mice **A.** PARP-1 deletion improved hyperglycemia-induced cardiac remodeling. **B.**, **C.**, **D.** Hyperglycemia increased the protein expression of nitrotyrosine and PARP-1. **E.** Hyperglycemia increased PARP-1 activity. Values are expressed as mean ± S.D. *P < 0.05 *vs*. control. DM: diabetic cardiomyopathy; si-PARP-1: PARP-1 siRNA; si-NC: negative control of PARP-1 siRNA.

There was no significant difference of heart rate and blood pressure among the three groups. STZ treatment increased the blood glucose and reduced body weight. Heart weight and the ratio of heart weight to body weight were significantly higher in diabetic mice than that in control mice. PARP-1 deletion reduced hyperglycemia-induced upregulation of heart weight and the ratio of HW/BW (Table 2). Then we detected the morphological structure of the hearts. The hematoxylin and eosin (H&E) staining showed that compared with control, diabetic mice hearts displayed structural abnormalities, but the hearts from the PARP-1 group did not show significant abnormality. Meanwhile, Compared with control mice, diabetic mice showed increased cardiomyocytes width, and PARP-1 gene deletion attenuated the enlarged cardiomyocytes (Figure 6A).

**Table 1:** Gene primers for RT-PCR

gene	forward(5′-3′)	reverse(5′-3′)
PARP-1	TTGAAAAAGCCCTAAAGGCTCA	CTACTCGGTCCAAGATCGCC
ICAM-1	TTGGAAGCCTCATCCG	CAATGTTGCGAGACCC
iNOS	GTTCTCAGCCCAACAATACAAGA	GTGGACGGGTCGATGTCAC
β-actin	TGGACATCCGCAAAGAC	GAAAGGGTGTAACGCAACTA

PARP-1, poly(ADP-ribose) polymerase 1; ICAM-1, intercellular adhesion molecule-1; iNOS, inducible nitric oxide synthase.

**Table 2:** General features of mice

	control(*n*= 10)	DM(*n*= 10)	PARP-1(*n*= 10)
SBP(mmHg)	112.9±6.21	108.2±4.94	112.9±8.95
DBP(mmHg)	77.8±5.63	79.4±5.27	78.6±6.05
HR(beats/min)	591.3±17.72	589.8±21.78	595.5±24.52
BG(mmol/l)	5.55±0.69	20.36±2.69*	21.47±2.41*
BW(g)	27.59±1.95	22.11±1.68^*^	23.02±2.19^*^
HW(mg)	120±3.82	130.7±3.17^*^	121.95±2.53^#^
HW/BW	4.38±0.24	5.66±0.26^*^	4.58±0.36^#^

DM, diabetes mellitus; SBP, systolic blood pressure; DBP, diastolic blood pressure; HR, heart rate; BG, blood glucose; BW, body weight; HW, heart weight.

* *p* < 0.05 *vs*. control;

# *p* < 0.05 *vs*.DM.

### Hyperglycemia increased nitrotyrosine as well as PARP-1 expression and activity

Compared with the control mice, hyperglycemia could increase the protein expression of nitrotyrosine, with an increase of PARP-1 expression and activity in DM mice. These results suggested that hyperglycemia induced oxidative damage and increased PARP-1 expression and activity (Figure 6B-6E).

### PARP-1 deletion reduced inflammatory response in mice

**Figure 7:**
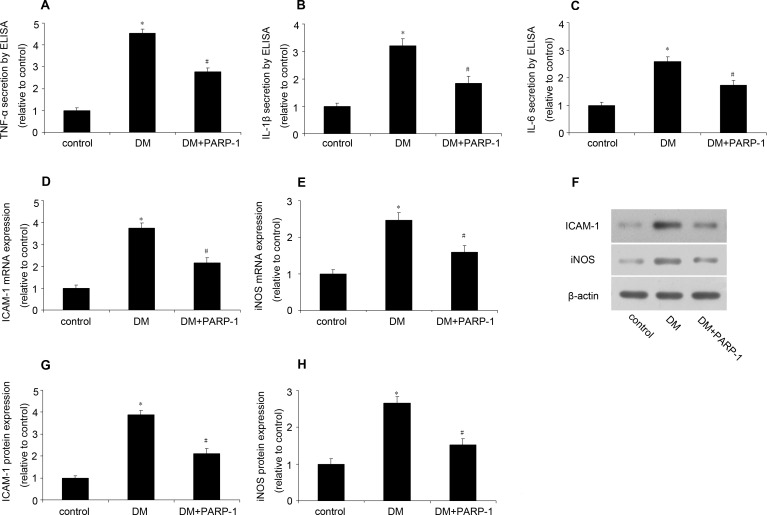
PARP-1 deletion reduced hyperglycemia-induced inflammatory response in mice In mice, the plasma concentration of TNF-α, IL-1β and IL-6 was determined by ELISA, the expression of ICAM-1 and iNOS was assessed by RT-PCR and western blotting analysis. **A.**, **B.**, **C.** PARP-1 deletion reduced hyperglycemia-upregulated plasma concentration of TNF-α, IL-1β and IL-6. **D.**, **E.** PARP-1 deletion reduced hyperglycemia-upregulated the mRNA expression of ICAM-1 and iNOS. **F.**, **G.**, **H.** PARP-1 deletion reduced hyperglycemia-upregulated the protein expression of ICAM-1 and iNOS. Values are expressed as mean ± S.D. *P < 0.05 *vs*. control. ^#^*P* < 0.05 *vs*. DM. DM: diabetic cardiomyopathy.

The concentration of TNF-α, IL-1β and IL-6 in plasma was assessed by ELISA. As shown in Figure 7A-7C, hyperglycemia could significantly increase these cytokines secretion compared with the control, while PARP-1 deletion reduce it. Meanwhile, hyperglycemia increased the mRNA and protein expression of ICAM-1 and iNOS, while PARP-1 deletion reduced hyperglycemia-induced ICAM and iNOS expression (Figure 7D-7H). These results suggested that PARP-1 deletion could reduce hyperglycemia-induced inflammatory response in mice.

### PARP-1 deletion reduced apoptosis partly through activation IGF-1R/Akt pathway in mice

**Figure 8:**
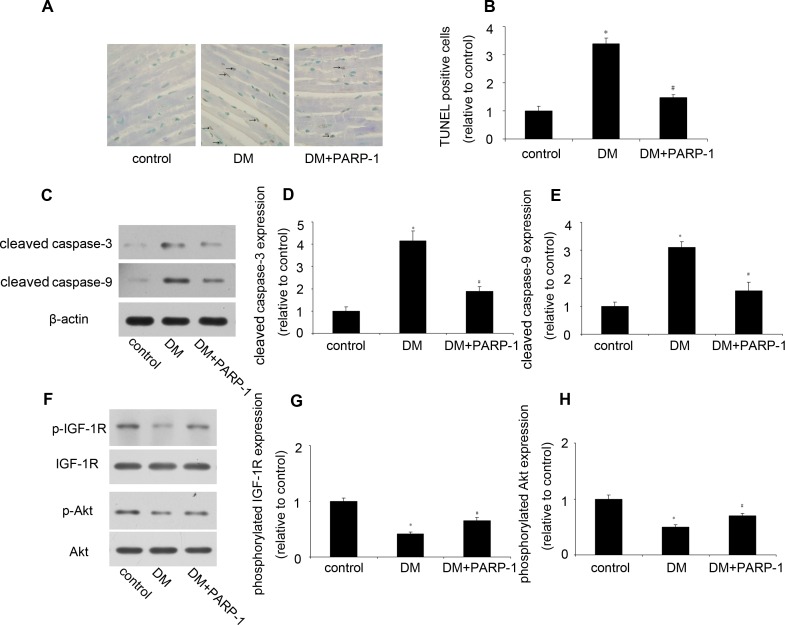
PARP-1 deletion reduced hyperglycemia-induced cardiomyocyte apoptosis **A.**, **B.** PARP-1 deletion reduced hyperglycemia-induced cardiomyocyte apoptosis. **C.**, **D.**, **E.** PARP-1 deletion reduced hyperglycemia-upregulated the protein expression of cleaved caspase-3 and caspase-9. **F.**, **G.**, **H.** PARP-1 deletion increased hyperglycemia-reduced the phosphorylation of IGF-1R/Akt. Values are expressed as mean ± S.D. *P < 0.05 *vs*. control. ^#^*P* < 0.05 *vs*. DM. DM: diabetic cardiomyopathy.

Then we detected cell apoptosis by TUNEL in mice heart. In control mice hearts, TUNEL-positive cells was seldom identified, but numerical TUNEL-positive cells were observed in DM mice. However, PARP-1 deletion significantly decreased the number of apoptotic cardiomyocytes (Figure 8A and 8B). Then we investigated the underlying mechanism, compared with the control mice, the protein expression of cleaved caspase-3 and caspase-9 was upregulated in DM mice, while PARP-1 deletion reduced their expression (Figure 8C-8E). Moreover, as shown in Figure 8F to 8H, hyperglycemia reduced p-IGF-1R and p-Akt expression, while PARP-1 deletion could increase their expression, which suggested that PARP-1 deletion protected cardiomyocytes from apoptosis partly through activation of IGF-1R/Akt pathway.

## DISCUSSION

Diabetic cardiomyopathy (DCM) is characterized by complex changes in the mechanical, biochemical, and structural properties of the heart, which may be responsible for the development of cardiac dysfunction. However, the precise mechanism of DCM has not been fully investigated. In our experiment, *in vitro*, we found that high glucose (HG) increased oxidative stress and induced DNA damage, with an increase of PARP-1 expression and activity. PARP-1 inhibition reduced HG-induced inflammatory response, including the secretion of TNF-α, IL-1β and IL-6 and the expression of ICAM-1 and iNOS. Moreover, PARP-1 inhibition decreased HG-induced cardiomyocytes apoptosis through downregulation of cleaved caspase-3 and caspase-9 as well as activation of IGF-1R/Akt pathway. *In vivo*, PARP-1 deletion restored cardiac function and alleviated cardiac remodeling. PARP-1 deletion could also reduce hyperglycemia-induced inflammatory response and cardiomyocyte apoptosis in mice. These results suggested that PARP-1 might be a therapeutic target for the treatment of DCM.

Recent studies have suggested that hyperglycemia induced oxidative damage plays a critical role in the early onset of DCM. Consistent with the theory, significant expression of nitrotyrosine has been found in myocardial biopsy samples from diabetic patients and in a mouse model of STZ-induced diabetes [14, 28]. Nitrotyrosine, as a marker of peroxynitrite formation, has been found in a number of disease-affected tissues or organs, such as atherosclerotic plaque, ischemia/reperfusion hearts, and artery after ballon injury [29]. It has been demonstrated that PARP-1 can be activated by high glucose or hyperglycemia in several cell lines, such as endothelial cells and umbilical endothelial cells [30–32]. In our experiment, we found that high glucose or hyperglycemia could increase oxidative stress and induce DNA damage assessed by comet assay. PARP-1 overactivation can contribute to the development of diseases *via* two mechanisms: by driving the cell into energy crisis and a state of dysfunction and by catalyzing the activation of pro-inflammatory pathways. Previous study has demonstrated that PARP-1 can be activated byin STZ-induced diabetes and plays a critical role in the development of myocardial and endothelial cardiovascular dysfunction in diabetes rats and mice [33]. Pharmacological inhibition or genetic deletion of PARP-1 is therapeutically effective in cardiovascular, inflammatory, vascular, and neurodegenerative diseases [34–36]. In the present study, we found that HG or hyperglycemia could increase the expression and activity of PARP-1 and induce inflammatory response, while PARP-1 inhibition significantly reduced the inflammatory response, including the secretion of TNF-α, IL-1β and IL-6 and the expression of ICAM-1 and iNOS. The precise mechanism needs further investigation. Meanwhile, PARP-1 deletion restored cardiac function and remodeling in mice.

Myocardial cell death, hypertrophy, and interstitial and perivascular fibrosis at late phase are the most frequently proposed mechanisms to explain cardiac changes in DCM. As cardiomyocytes rarely proliferate in adult human heart, the loss of cardiac muscle cells would eventually lead to cardiac dysfunction. PARP-1 inhibition can reduce mediates structural alterations of hearts in DCM [37]. In our experiment, HG or hyperglycemia induced cardiomyocyte apoptosis, while PARP-1 inhibition or deletion could significantly reduce the number of apoptotic cells, with downregulation of cleaved caspase-3 and caspase-9.

Insulin-like growing factor-1 (IGF-1) overexpression has been shown to attenuate cardiomyocytes apoptosis and necrosis in transgenic mice [38, 39]. The type 1 IGF receptor (IGF-1R), a member of a family of transmembrane tyrosine kinases, can bind IGF-1 with high affinity and initiate physiological function [40]. Once the ligand interacts with the IGF-1R α-subunit, tyrosine residues in the intracellular, β-subunit will become phosphorylated [41]. Phosphorylation of IGF-1R can activate Akt by a cascade signaling pathway, eliciting a repertoire of cellular responses including proliferation, and the protection of cells from death [42].

In summary, these results provide a deeper understanding of the regulatory role of PARP-1 in diabetic heart inflammation and apoptosis, indicating that PARP-1 inhibition may be a feasible strategy for treating diabetic heart injury and cardiomyopathy.

## MATERIALS AND METHODS

All experiments were performed in compliance with the Guide for the Care and Use of Laboratory Animals (NIH Publication No. 85-23, revised 1996) and were approved by the Ethics Committee of Shandong University.

### STZ-induced model of diabetes

To induce diabetes, male C57BL/6 (WT) mice or PARP-1^−/−^ mice (10 weeks old, Jackson Laboratories, ME, USA) were treated with STZ (sigma, 50mg/kg in citrate buffer, pH 4.5) by intraperitoneal injection for 5 consecutive days [26], while the control animals (male C57BL/6 mice) received the same volume of citrate buffer. Mouse tail vein blood glucose levels were monitored by analysis with the Roche Accu-Chek Active blood glucose monitor. The mice with a fasting blood glucose concentration >11.1 mmol/l were considered diabetic. Mice were housed in a pathogen-free animal care facility and allowed free access to food and water. PARP-1^−/−^ mice were genotyped by PCR (Supplementary Figure 1).

### Echocardiography

Cardiac diameter and function were measured by use of the Vevo 770 imaging system (VisualSonics, Toronto, Canada). Left ventricular (LV) ejection fraction (LVEF), fractional shortening (FS), ratio of early to late mitral inflow velocity (E/A), and left ventricular end-diastolic dimension (LVEDd) were measured. All measurements were performed by the same observer and were the average of five consecutive cardiac cycles. Heart rate, systolic blood pressure, and diastolic blood pressure were measured with a noninvasive tail-cuff system (Softron BP-98A; Softron, Tokyo, Japan) as described previously [3].

### Tissue harvest and H&E staining

Mice were sacrificed with intraperitoneal injection of ketamine/xylazine solution (80:8 mg/kg) after 12 weeks. After perfused with phosphate buffered saline (PBS), mice hearts were harvested and frozen in liquid nitrogen and stored at −80°C for molecular biology analysis or fixed in 4% polyformaldehyde for staining. The hearts morphologic features were assessed by use of a microscope. Paraformaldehyde (4%) fixed hearts were bisected transversely at the midventricular level, embedded in paraffin, and cut into 5um sections. Serial cryosections were stained with hematoxylin and eosin (H&E, Sigma-Aldrich, MO, USA).

### TUNEL

Apoptosis of mice heart tissue was detected by use of ApopTag Plus Peroxidase In Situ Apoptosis Detection Kit (Millipore, MA, USA) according to the manufacturer's protocol. The number of TUNEL-positive cardiomyocyte nuclei and the total cardiomyocyte nuclei in each sight were counted. The ratio of apoptotic cardiomyocytes was calculated by dividing the number of TUNEL-positive cardiomyocyte nuclei by the number of total cardiomyocyte nuclei.

### Cell culture and stimulation

H9c2 cardiomyocytes (ATCC, USA) were cultured in Dulbecco's modified Eagle medium (DMEM, ScienCell, CA, USA) supplemented with 10% fetal bovine serum, 100U/ml penicillin and 100ug/ml streptomycin with 5% CO_2_ at 37°C. Subcultures were performed with trypsin-EDTA. *In vitro* experiments, cells were treated with normal glucose (control, 5.5 mmol/l glucose), mannitol (5.5 mmol/l glucose plus 24.5 mmol/l mannitol) or high glucose (HG, 33mmol/l glucose). To inhibit PARP-1 expression, H9c2 cells were transiently transfected with PARP-1 siRNA or negative control (GenePharma, Shanghai, China) in Optimem Medium (Invitrogen, CA, USA) with use of Lipofectamine 2000 (Invitrogen). Experiments were performed 24h after transfection.

### ROS and superoxide union (O_2_^−^) assay

Cellular ROS levels were determined by use of the Reactive Oxygen Species Assay Kit (Beyotime, Haimen, China) according to the manufacturer's instructions. Briefly, cells in 24-well plates were incubated with normal glucose, mannitol or high glucose, then washed twice with PBS and incubated with 2′,7′-dichlorofluorescein diacetate (DCFH-DA) for 30 min at 37°C. The ROS level was determined on the oxidative conversion of DCFH-DA to fluorescent dichlorofluorescein on reaction with ROS in cells. For cellular O_2_^−^ assay, cells were incubated with 5μM dihydroethidium (DHE) for 30 min at 37°C and then under went a drop of Prolong Gold antifade reagent with DAPI staining for 5 minutes. Images were acquired with a Zeiss LSM710 confocal microscope (Zeiss, Germany).

### Comet assay

The DNA damage of H9c2 cells was assessed by using comet assay kit (Trevigen, MD, USA). Cells were harvested by scraping in cold PBS, centrifuged at 1000 g and re-suspended in PBS to 10^5^ cells/ml. The cell suspension (50 μl) was quickly mixed with 500 μl Low Melting Point agarose, then 50 μl of the mixture was dropped and spreaded over sample area, and allowed to gel at 4°C. Slides were submerged in lysis solution for 40 minutes at 4°C and then placed in Alkaline Unwinding Solution to incubate for 40 minutes at room temperature. The slides were then placed under freshly prepared electrophoresis buffer and electrophoresed at 21V for 30 min at 4°C. Then the slides were immersed in dH_2_O, followed by 70% alcohol for 5 min and then allowed to air dry. The slides were stained by immersion in SYBR Green I and photographed under a fluorescence microscope. Slides were scored using CometScore software v1.5 (TriTek Corporation, Summerduck, VA).

### ELISA

The levels of TNF-α, IL-1β and IL-6 in the cultured cell medium and mouse plasma were determined by use of the ELISA kit (Uscn Life Science Inc., Wuhan, China). Mean absorbance was assessed in duplicate and all operations were performed at room temperature. The color reaction was detected by use of Varioskan Flash multifunction plate reader (Thermo Scientific, Rockford, USA).

### Real-time quantitative PCR

Cellular and tissue RNA was extracted by using Trizol reagent (Invitrogen). The cDNA was analyzed by real-time RT-PCR with SYBR Green Supermix (Bio-Rad Laboratories, CA, USA). The primers for PARP-1, ICAM-1, iNOS and β-actin were shown in Table 1. Amplification, detection, and data analysis were performed by the use of the iCycler real-time PCR system (Bio-Rad Laboratories). Each sample was analyzed in triplicate, and expression was normalized to β-actin. The gene expression was obtained by the 2-ΔΔCt calculation method.

### Western blotting analysis

H9c2 cells and mice hearts were freshly harvested and homogenized in RIPA lysis buffer (Beyotime, Beijing, China), sonicated for 20s, and normalized with the BCA Protein Assay Kit (Beyotime). The protein was separated by 10% SDS/PAGE and then electro-transferred onto nitrocellulose membrane (Millipore, MA, USA). After blocked with 5% non-fat milk for 2hrs, the nitrocellulose membrane was washed in Tris-Buffered Saline Tween-20 (TBS-T) for three 10mins and incubated with gentle agitation at 4°C overnight with the primary antibodies to PARP-1 (1:2000, sigma, CA, USA), ICAM-1 (1:500; Santa Cruz Biotechnology, CA, USA), iNOS (1:1000, cell signaling technology, MA, USA), cleaved caspase-3 (1:1000, cell signaling technology), cleaved caspase-9 (1:1000, cell signaling technology), IGF-1R (1:1000, cell signaling technology), p-IGF-1R (1:1000, cell signaling technology), Akt (1:1000, cell signaling technology), p-Akt (1:1000, cell signaling technology), nitrotyrosine (1:2000, cayman, MI, USA), β-actin (1:1000, Cell Signaling Technology) followed by washes with TBS-T and subsequent incubation with appropriate horseradish peroxidase-conjugated secondary antibody for 2hrs at room temperature. Labeled protein was visualized through enhanced chemiluminescence (Millipore).

### Flow cytometry

H9c2 cells were plated on 6-well plates. After treatment, cells were harvested and stained with Annexin-V and Propidium Iodide (PI) for 10 min at room temperature in the dark. The cell apoptosis was measured by flow cytometry (FACS Calibur, BD, NJ, USA) within 30mins.

### PARP-1 activity assay

PARP-1 activity was assayed based on the incorporation of biotinylated ADP-ribose onto histone proteins (R&D Systems, MN, USA). Cell or tissue lysates containing 50 ug of protein were loaded into a 96-well plate coated with histones and biotinylated polyADP-ribose, allowed to incubate for 1hrs, treated with strep-HRP, and read at 450 nm in a spectrophotometer (Thermo Scientific).

### Statistical analysis

Values are presented as means±SD. SPSS 16.0 was used for statistical analysis. Statistical significance between two measurements was determined by the two-tailed unpaired Student's t test, and among groups, it was determined by one-way ANOVA. A value of *p* < 0.05 was considered statistically significant.

## SUPPLEMENTARY MATERIALS FIGURES


