# Supplementary material for: Poly(ADP-ribose) polymerase 1 inhibition protects cardiomyocytes from inflammation and apoptosis in diabetic cardiomyopathy
Source: Oncotarget. 2016 Mar 24;7(24):35618–31. doi: 10.18632/oncotarget.8343 (PMC5094949; doi:10.18632/oncotarget.8343)
Supplement: Supplementary file 1 [file oncotarget-07-35618-s001.pdf]

Poly(ADP-ribose) polymerase 1 inhibition protects cardiomyocytes from inflammation and apoptosis in diabetic cardiomyopathy

Supplementary Material

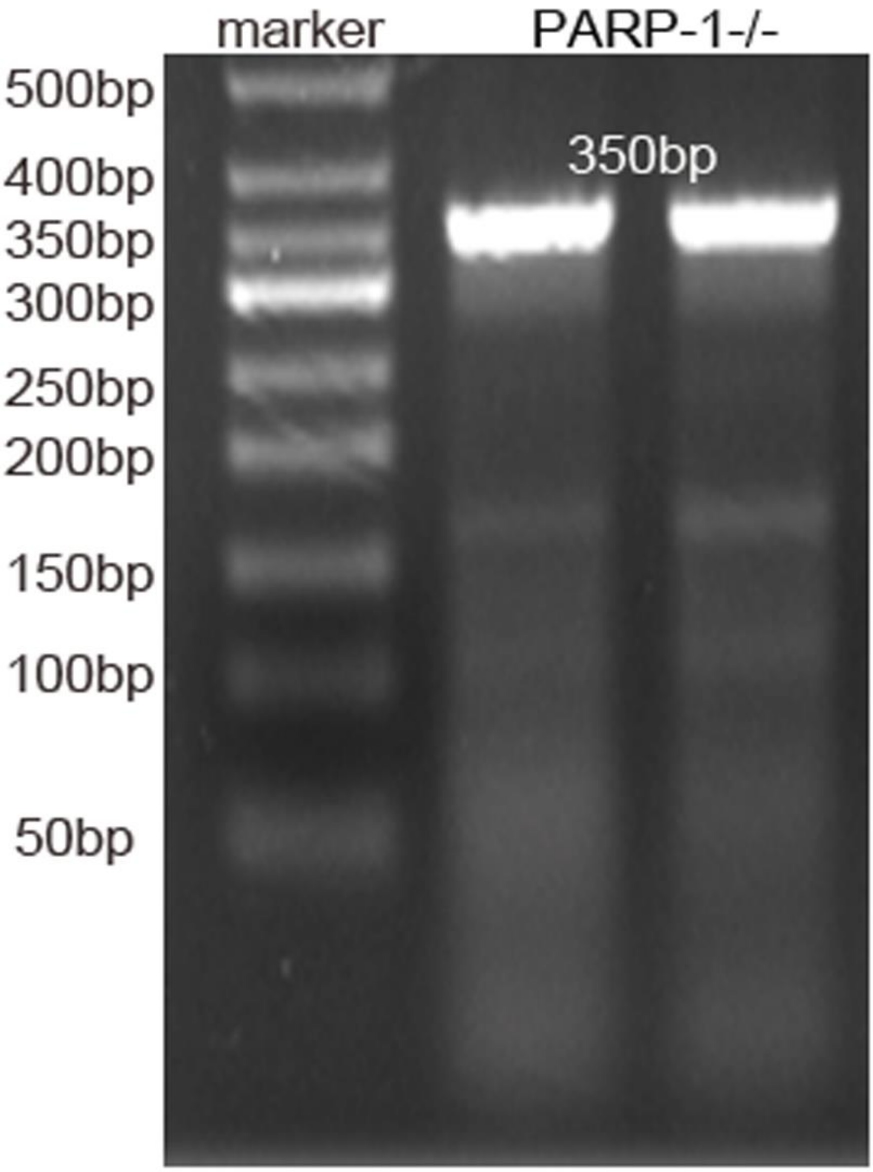

Supplementary Figure 1

Supplementary Figure 1: PARP-1 gene knockout mice were genotyped by RT-PCR.

The mice DNA was extracted from tail by use of DNA extraction kit (TransGen

Biotech, Beijing, China) according to the manufacturer's protocol and analyzed by RT-PCR. The primers were as follows: 5-CCAGCGCAGCTCAGAGAAGCCA-3 (Wild type); 5-CATGTTCGATGGGAAAGTCCC-3(Wild type); 5-AGGTGAGATGACAGGAGATC-3 (Mutant Reverse). Wild type: 112bp only; PARP-1$^{-/-}$: 350bp only; heterozygote: 112bp and 350bp.

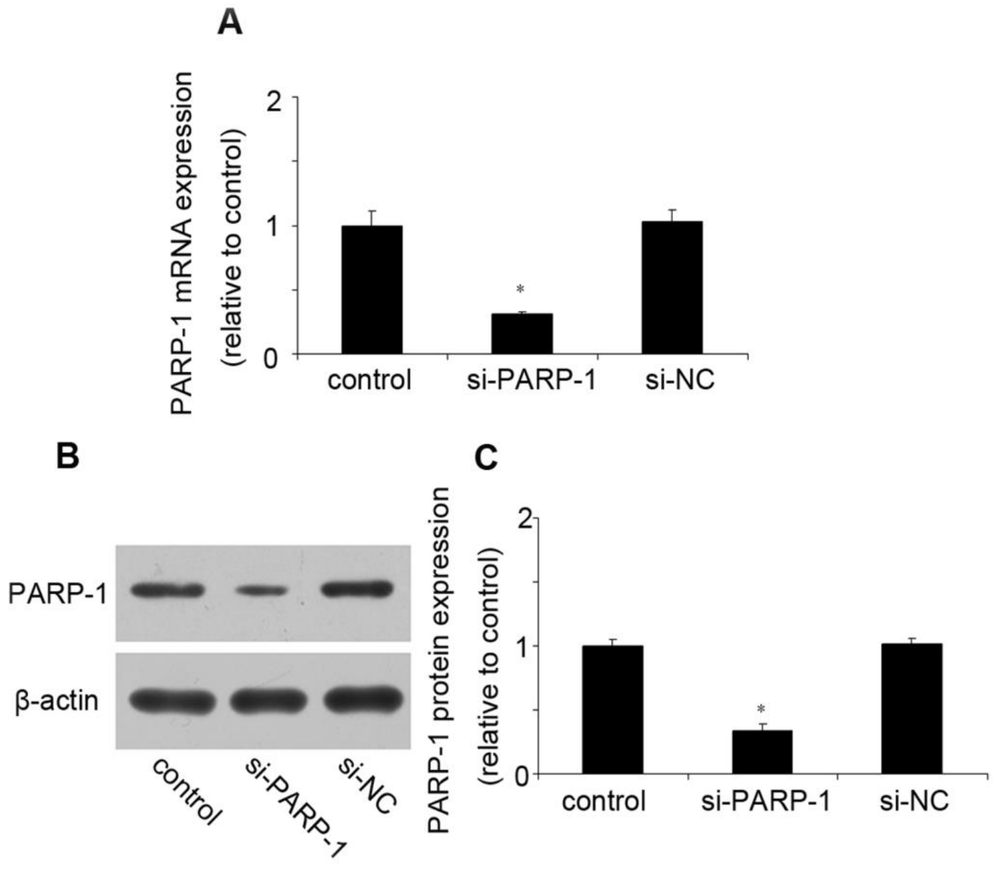

Supplementary Figure 2: PARP-1 expression was inhibited by siRNA.

(A) PARP-1 mRNA expression was inhibited by siRNA as assessed by RT-PCR; **(B, C)** PARP-1 protein expression was inhibited by siRAN as assessed by western blotting analysis, while the negative control of siRNA had no effect. Values are expressed as mean ± S.D. **P*<0.05 vs. control. Si-PARP-1: PARP-1 siRNA; si-NC: the negative control of PARP-1 siRNA.
